# Consequences of imprecision in fetal fraction estimation on performance of cell‐free DNA screening for Down syndrome

**DOI:** 10.1002/pd.6126

**Published:** 2022-03-11

**Authors:** Fredrik Persson, Howard S. Cuckle

**Affiliations:** ^1^ Vanadis Diagnostics PerkinElmer Inc. Sollentuna Sweden; ^2^ Faculty of Medicine Tel Aviv University Tel Aviv Israel

## Abstract

**Background:**

There is a significant variability in reported fetal fraction (FF), a common cause for no‐calls in cell‐free (cf)DNA based non‐invasive prenatal screening. We examine the effect of imprecision in FF measurement on the performance of cfDNA screening for Down syndrome, when low FF samples are classified as no‐calls.

**Methods:**

A model for the reported FF was constructed from the FF measurement precision and the underlying true FF. The model was used to predict singleton Down syndrome detection rates (DRs) for various FF cut‐offs and underlying discriminatory powers of the test.

**Results:**

Increasing the FF cut‐off led to slightly increased apparent DR, when no‐calls are excluded, and an associated larger decrease in effective DR, when no‐calls are included. These effects were smaller for tests with higher discriminatory power and larger as maternal weight increased.

**Conclusions:**

Most no‐calls due to a low reported FF have a true FF above the cut‐off. The discriminatory power of a test limits its effective DR and FF precision determines the tradeoff between apparent and effective DR when low FF is used to discard samples. Tests with high discriminatory power do not benefit from current FF measurements.

## Key points

What is already known about this topic?

- Fetal fraction (FF) is often considered to be a crucial quality control parameter for interpretation of cell free DNA based non‐invasive prenatal testing (NIPT)
- There is a large variability in the measurement of FF for single samples
- A large fraction of test non‐reportable results (no‐calls) are due to a too low reported FF

What does this study add?

- This article presents the consequences of the high variability in FF measurements in the context of screening NIPT test performance
- For tests with a high discriminatory power, discarding samples based on too low reported FF leads to a slight apparent increase in NIPT performance metrics but at a relatively large expense of unnecessary anxiety, clinical and financial burden of additional counseling and follow‐up procedures

## 1 INTRODUCTION

Over the last decade a large number of women have had a cell‐free (cf)DNA screening test for fetal aneuploidy. So far, most tests have been carried out in the private sector, but increasingly public health screening programs are being established, whereby women are offered a cfDNA test. In light of this, many professional societies and organizations have published recommendations or practical guidelines associated with the utility of cfDNA as a screening assay for fetal aneuploidies, as well as recognizing the importance of ‘no‐call’ results that commonly are excluded from reported performance metrics.^1^, ^2^, ^3^, ^4^

These cfDNA tests rely on the presence of a cfDNA fraction in maternal plasma that originates from the placenta, acting as a proxy for the fetus. The amount of feto‐placental cfDNA compared to the total cfDNA is commonly referred to as the fetal fraction (FF) and, in the first trimester, averages around 10%–11% with a wide range among individuals being tested.^5^, ^6^, ^7^ All cfDNA tests rely on the ratio between a chromosome of interest and a reference chromosome or set of chromosomes. This ratio can be normalized so that euploid samples have, on average, a ratio of 1, thus in Down syndrome pregnancies, the average ratio would be [3FF + 2(1 ‐ FF)]/2 or 1 + FF/2 since the fetus has three copies of chromosome 21 while the mother has two and both have two copies of the reference chromosomes.^8^

In general, the ability of the test to distinguish aneuploid from euploid pregnancies (‘discriminatory power’) is dependent on the overlap of chromosomal ratios between affected and unaffected pregnancies; and for the individual sample it is also dependent on the FF. This has led to the notion that FF should be routinely quantified and the cfDNA result only reported when it is above a pre‐set, or dynamic, limit, with the remainder classified as no‐calls.^1^ Direct quantification of FF can sometimes be done by measuring either the Y‐chromosome, when the fetus is known to be a singleton male,^9^, ^10^ or the affected chromosome when it is known to be trisomic.^8^ But for routine testing, indirect quantification is needed. Several approaches have been described, including those based on fragment size distributions,^11^ nucleosome profiles,^12^ and Single Nucleotide Polymorphisms (SNPs).^13^, ^14^ Comparison of direct and indirect quantification shows that FF measurements for individual samples are inaccurate^8^, ^15^ due to its considerable imprecision; with a standard deviation (SD) in six published studies ranging from 1.3% to 3.4%.^16^ The consequences of this FF imprecision have generally not been reported, except in samples from non‐pregnant women.^17^, ^18^

The imprecision of FF measurements, together with a lack of standardization, has led some to question the use of this metric in deciding whether to report a cfDNA result or not.^19^, ^20^ In this paper, modeling is used to examine the effect of FF imprecision on the no‐call rate and consequently the Down syndrome detection rate (DR) among singleton pregnancies in a universal cfDNA screening program.

## 2 METHODS

A model was constructed for the joint frequency distribution of FF values in the absence of FF assay imprecision (truFF) and values estimated by a given assay method (estFF). The model comprises a log Gaussian distribution of truFF and a marginal Gaussian distribution of estFF for a given truFF. There are three model parameters—mean, SD of log(truFF) and SD of the FF assay—which are the same in both Down syndrome and unaffected pregnancies.

The mean and SD of log (truFF) were derived from a published study of 10,698 singleton pregnancies tested prospectively at 10–14 weeks gestation, which included 10,472 unaffected, 160 Down syndrome, 50 Edwards syndrome and 16 Patau syndrome pregnancies.^5^ The reported FF was shown to follow an approximately log Gaussian distribution with a heavier lower tail which would be expected if FF assay imprecision is additive rather than proportional. Therefore, to minimize the influence of the FF assay, the mean of log(truFF) was estimated from the median (11.0%) and the SD from the inter‐quartile range (8.3%–14.4%),^21^ yielding log(truFF) values of −0.959 and 0.179, for the mean and SD respectively. There was no difference in the reported FF distribution between the Down syndrome and unaffected pregnancies according to statistical hypothesis testing (*p* = 0.97, two‐tailed *t*‐test) and a multi‐variate regression analysis.^5^

The SD of the same FF assay was derived from a published study of FF measurement in 47,512 singleton pregnancies with male fetuses, tested after 10 weeks gestation.^14^ A published digital analysis of a plot between the reported value (estFF) and that from the Y‐chromosome, representing the truFF, found that the assay imprecision followed a Gaussian distribution with an SD of 1.6%.^16^

The model was used to simulate estFF and truFF in one million data points. Goodness of fit was assessed using the reported FF values in the 10,472 unaffected pregnancies provided by Revello et al.^5^

The model was used to estimate the no‐call rate for different FF cut‐offs as well as the proportion of truFF values below the cut‐off. Additionally, both the apparent (app)DR, excluding no‐calls, and the effective (eff)DR, when affected no‐calls are included as screen negative, were evaluated for Down Syndrome pregnancies. This was done for an idealized cfDNA test assumed to have a hard limit of detection (hLoD) defined as a truFF value above which all Down syndrome results are true‐positive, and below which all are false‐negative. Tests with four hLoD values were used in the analysis (2%, 3%, 4% and 5%) and samples that are classified as no‐call for technical reasons were not considered. The effDR and appDR of these tests were compared with the intrinsic (int)DR when no FF cut‐off is applied.

Fetal fraction is negatively correlated with maternal weight^5^, ^6^ consequently reducing Down syndrome detection in heavier women. This was investigated by updating the parameters for the truFF distribution using a subset of the 10,472 unaffected pregnancies from Revello et al.^5^ with a maternal weight between 55 and 65 kg (4,184 samples), while keeping FF assay imprecision the same. The mean of truFF for a given maternal weight was taken as the median reported FF for this subset, shifted according to a log‐linear regression analysis showing a decrease of log(FF) by 0.00529 per kg (95% CI: 0.00505–0.00554). The truFF SD was derived under the assumption that the coefficient of variation is unaltered by maternal weight.

## 3 RESULTS

**FIGURE 1:**
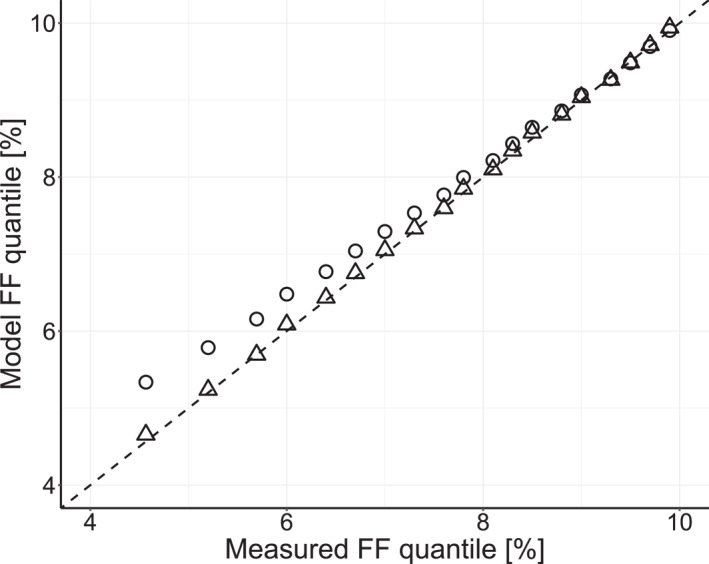
Goodness‐of‐fit comparing selected quantiles of modeled true fetal fraction (FF), without (○) and with (△) the inclusion of measurement imprecision (SD = 1.6%), against the reported FF

Figure 1 shows an almost perfect agreement in estFF quantiles between the observed reported FF and the model predicted FF, confirming the model assumptions and derived parameters.

Table 1 shows, for selected FF cut‐off levels ranging from 1% to 5%, the model predicted proportion of samples with truFF and estFF below the cut‐off. In each case the proportion for estFF is much higher than for truFF, thus the vast majority of samples classified as no‐call for having estFF below a cut‐off in this range would have a truFF above the cut‐off. This proportion of erroneously classified samples, for different FF cut‐offs, is also shown in Table 1.

**TABLE 1:** Model predicted proportion of samples with no‐call results according to the FF cut‐off for both the truFF and estFF

Samples	FF cut‐off				
	1%	2%	3%	4%	5%
truFF < cut‐off	0.0%	0.0%	0.1%	0.7%	2.8%
estFF < cut‐off	0.1%	0.3%	1.0%	2.5%	5.2%
estFF < cut‐off & truFF ≥ cut‐off	>99.9%	99.5%	95.0%	82.2%	64.8%

Table 2 compares the model‐predicted appDR and effDR for four cfDNA tests according to different FF cut‐off levels. When no FF cut‐off is applied, both the appDR and effDR will be equal and reflect the intDR. For all tests with a hLoD ≤4%, a minor gain in appDR is associated with a substantially larger loss in effDR, reflecting that the majority of samples converted to no‐calls had a sufficiently high truFF to be called correctly. For a test with a high discriminatory power (hLoD = 2% or 3%), a 4% FF cut‐off would give an increase in appDR *<*0.1% and a decrease in effDR of 2.5%. In addition, it should be noted that even having a FF cut‐off perfectly matched with the hLoD, the appDR is less than 100%, reflecting the fraction of samples with truFF *<* 4% that gets misclassified with an estFF ≥ 4%, resulting in false negative results.

**TABLE 2:** Model predicted Down syndrome effDR and appDR according to hLoD and no‐call FF cut‐off applied to estFF

hLoD (intDR)	FF cut‐off							
	2%		3%		4%		5%	
	effDR	appDR	effDR	appDR	effDR	appDR	effDR	appDR
2% (>99.9%)	99.7%	>99.9%	99.0%	>99.9%	97.5%	>99.9%	94.8%	>99.9%
3% (99.9%)	99.6%	99.9%	99.0%	>99.9%	97.5%	>99.9%	94.8%	>99.9%
4% (99.3%)	99.1%	99.4%	98.6%	99.6%	97.3%	99.7%	94.6%	99.9%
5% (97.2%)	97.1%	97.4%	96.8%	97.8%	95.9%	98.4%	93.8%	99.0%

Abbreviations: DR, detection rate; FF, fetal fraction; hLoD, hard limit of detection.

**FIGURE 2:**
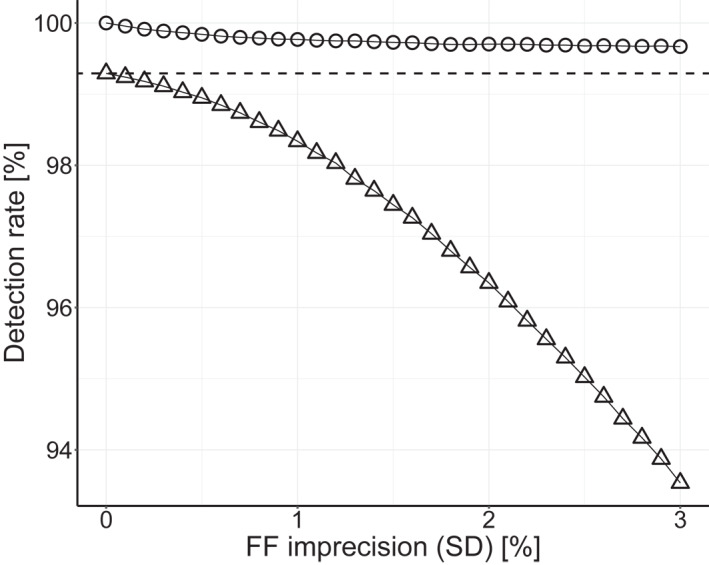
Model predicted cfDNA Down syndrome detection rates: appDR (○) and effDR (△) according to fetal fraction (FF) imprecision, for a test with hard limit of detection of 4% and a 4% FF cut‐off. The intDR is indicated by the dashed black line

Figure 2 shows the effect of varying FF assay imprecision on a test with hLoD 4% and matching 4% FF cut‐off. While the appDR does not decrease much with increasing imprecision, the effDR reduces substantially.

**FIGURE 3:**
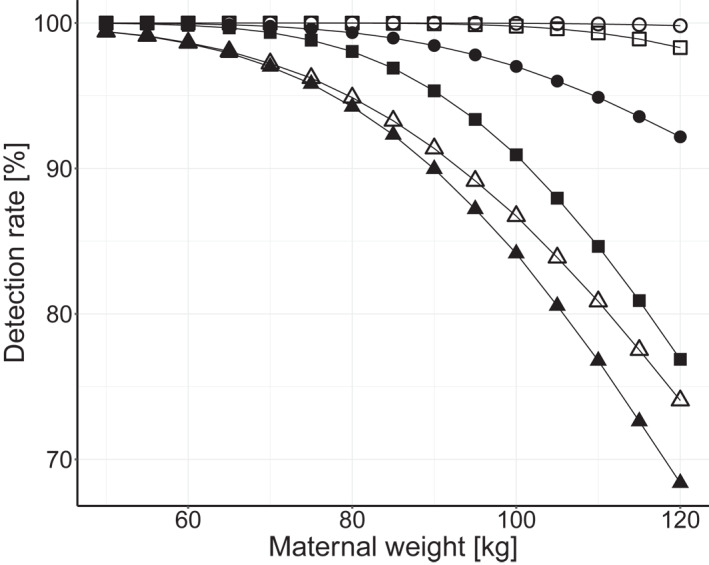
Model predicted cfDNA Down syndrome detection rates: appDR (○/●), effDR (△/▲) and intDR (□/■), according to maternal weight, for tests with hard limit of detection of 2% (open symbols) and 4% (closed symbols) and a 4% fetal fraction cut‐off

Table 3 shows the corresponding DRs for women with a maternal weight of 100 kg. Both the appDR and effDR are lower and the separation between them is increased as compared to Table 2. Figure 3 shows, for tests with hLoD of 2% and 4%, and a FF cut‐off 4%, the effect of maternal weight on the intDR, effDR and appDR.

**TABLE 3:** Model predicted Down syndrome effDR and appDR according to hLoD and no‐call FF cut‐off applied to estFF, for pregnancies with a maternal weight of 100 kg

hLoD (intDR)	FF cut‐off							
	2%		3%		4%		5%	
	effDR	appDR	effDR	appDR	effDR	appDR	effDR	appDR
2% (99.8%)	98.3%	99.9%	95.1%	>99.9%	88.6%	>99.9%	78.9%	>99.9%
3% (97.5%)	96.5%	98.1%	94.5%	99.2%	88.4%	99.7%	78.8%	99.9%
4% (90.9%)	90.5%	92.0%	89.5%	94.0%	86.5%	97.6%	78.2%	99.1%
5% (80.2%)	80.1%	81.4%	79.7%	83.8%	78.6%	88.7%	75.2%	95.3%

Abbreviations: DR, detection rate; FF, fetal fraction; hLoD, hard limit of detection.

## 4 DISCUSSION

The model developed in this study demonstrates the effect of imprecision in FF measurement on cfDNA screening performance for Down syndrome in singleton pregnancies. When a FF cut‐off is used to classify results as no‐calls the performance is determined by both the discriminatory power of the cfDNA test itself and the imprecision of FF measurement. The model shows that a very large proportion of these no‐calls actually have a true FF above the cut‐off, and the associated effects on Down syndrome effDR and appDR. It also shows how the Down syndrome DR is negatively affected by both poor discriminatory power and FF imprecision. For a given discriminatory power, the effect of the imprecision of FF measurement may not be very apparent in the detection rate after excluding no‐calls, as in most publications, but has a substantial effect when the no‐calls are classified as screen‐negative. The aforementioned effects are even greater when screening obese women.

The model parameters relating to the distribution of FF were derived from a series of women having cfDNA screening at 10–14 weeks gestation in England, which may not be entirely representative of other locations. However, in a multivariate regression analysis the only statistically significant variables relating to FF that might differ between localities were maternal age, body‐mass index, South Asian ethnicity and assisted conception.^5^ Both the maternal age and South Asian effects were very small; assisted reproduction accounts for less than 5% of births in most localities; and effects from maternal weight on the Down syndrome detection rate was evaluated in this paper (Table 3 and Figure 3).

Similarly, the model parameter relating to FF measurement imprecision represents a single FF assay.^14^ However, this value, an SD of 1.6%, is consistent with the range of SDs, 1.3%–3.4%, reported or derived in a meta‐analysis using various FF assays.^8^, ^11^, ^12^, ^16^, ^22^, ^23^ Moreover, the effect of this SD on the Down syndrome detection rate was evaluated (Figure 2), suggesting that the effects in general are similar or worse with other approaches.

To predict Down syndrome detection rates in cfDNA tests with different discriminatory power, the model used hLoD, which assumes complete detection above and no detection below that true FF. In practice there is a continuous reduction in discriminatory power as FF approaches zero, rather than a sudden loss at a given true FF level. Nevertheless, the effects demonstrated using these simplified cfDNA tests would still apply to those used in practice. Similarly, the same effect of imprecision in the FF measurement is still valid when using a dynamic FF cut‐off in combination with, for example, sequencing coverage, instead of using a fixed cut‐off.^23^

The analysis assessed Down syndrome screening performance only by the detection rates and FF based no‐call rate, not by the false‐positive rate (FPR) or positive predictive value (PPV). That is because the FPR is generally, except for methods using the reported FF as a part of the risk assessment, independent of FF and very low for cfDNA tests, resulting in a high PPV. Since no‐calls based on technical reasons were not considered, the effective and intrinsic DRs should be considered upper estimates.

For the purposes of illustration, the effective Down syndrome detection rate was defined by categorizing no‐call results from affected pregnancies as screen negative. In clinical practice a repeat cfDNA test on a second blood sample is often offered after a no‐call result. However, not all women offered a retest submit one (50%–75%); and, only about two‐thirds of those receive a final result.^5^, ^24^, ^25^ In one study only 9% of unaffected pregnancies with a no‐call as a final result, that is after offered repeat testing, went on to have an invasive test.^5^ But if instead, as suggested by recent guidelines,^1^, ^3^ all no‐calls based on low FF lead to invasive testing the clinical FPR, defined as the rate of samples being forwarded to confirmatory testing, would increase by the no‐call rate and thus the clinically relevant PPV would be substantially decreased.^26^ Our model could be extended to include the proportion of no‐call results having a second sample tested. But there is insufficient published data on the discriminatory power of the second test, for which samples would be enriched for lower than average FF. In addition, redraw compliance can differ vastly between regions which would bias these results. Using information regarding local redraw compliance and resolve rates, the effects for that screening scenario can be estimated from the presented data.

The finding that a large proportion of samples designated as no‐call because of a reported FF below a given cut‐off actually have a FF above the cut‐off is consistent with a binomial counting model of Down syndrome Z‐scores, taking errors in FF measurements into account.^27^ In their model, Wright et al. noted that the curvilinear relationship between Down syndrome Z‐score and estFF was attributable to samples with erroneously underestimated truFF. Their model showed that >80% of samples with an estFF <4% were misclassified, in line with the 82.2% predicted here (Table 1).

When evaluating different cfDNA tests for use in a Down syndrome screening program, discriminatory power is the key performance indicator. However, many cfDNA tests do not provide sufficient information for users to judge this. It would be helpful if manufacturers would provide information indicating discriminatory power expressed either as the precision of the assay, that is, a measure of the variability in chromosomal ratio between unaffected samples, or as a version of the associated limit of detection.^28^, ^29^

The importance of having a test with a high discriminatory power becomes even more evident in populations with an increased maternal weight. For example, among women with a maternal weight of 100 kg it would require a test with an hLoD of 2% to have a DR equal to that of women with a maternal weight 60 kg having a test with an hLoD of 4%. Using the latter test for these women with a FF cut‐off of 6.5% would equalize the DR but result in a no‐call rate of 39%.

The analysis here is limited to Down syndrome. Other conditions, that tend to be associated with lower FF, such as Patau and Edwards syndrome,^5^ will have a shift in the relative increase in appDR and decrease in effDR, similar to what is seen for populations with an increased maternal weight. For other conditions that are screened, such as sex chromosome abnormalities, rare autosomal trisomies and micro‐deletion syndroms, it is still unknown whether they are associated with lower FF, or their decreased DR compared to Down syndrome is caused by an inherent lower discriminatory power.

Although there is no clear consensus regarding the merits of measuring and reporting FF,^4^ it has been suggested that cfDNA test reports should include an estimated FF.^1^ In light of both the high degree of imprecision in FF measurements and that cfDNA assays can have different performance for low FF samples, there is cause for this to be reconsidered. With many commercial cfDNA assays moving towards higher discriminatory power and dynamic FF cut‐offs to decrease FF based no‐calls, comparing estimated FF values to the widely accepted cut‐off of 4% can lead to misinformed decisions.

The current analysis also highlights the trade‐off between an increasing appDR and a decreasing effDR. For high discriminatory power (hLoD ≤ 3%) the decrease in effDR is more than 100‐fold greater than the very slight increase in appDR, suggesting that using low FF as a reason to classify results as no‐calls for these tests provides limited benefit and potentially even a disadvantage for the screening process as a whole considering the proportion of no‐calls that would be offered invasive testing. As has been clinically shown,^30^ such tests can have a very high intrinsic Down syndrome detection rate which would effectively be reduced by such classification. Moreover, not classifying results as no‐calls based on FF reduces the need for repeat cfDNA testing as well as invasive procedures with the associated anxiety and added clinical and financial burden.

## CONFLICT OF INTEREST STATEMENT

Howard S. Cuckle has received consulting fees and support for meeting attendance and/or traveling from PerkinElmer Inc.

## ETHICS STATEMENT

This study analyzes the effects of using fetal fraction to discard samples from reporting. The only clinical data used in this paper is previously published NIPT data on fetal fraction for the purpose of defining model parameters. No individual results are considered and for that reason, no approval of a medical ethical committee was required.

## Data Availability

The data that support the findings of this study are available from the corresponding author upon reasonable request.
